# Tomosynthesis-Guided Biopsy: A Troubleshooting Guide

**DOI:** 10.3390/diagnostics15030295

**Published:** 2025-01-27

**Authors:** Reve Chahine, Madiha Hijazi, Najwa Radwan, Ghina Berjawi, Lara Nassar

**Affiliations:** 1 Department of Diagnostic Radiology, University of Michigan, Ann Arbor, MI 48109, USA; chahinereve@gmail.com; 2 Department of Diagnostic Radiology, American University of Beirut Medical Center, Beirut 1107, Lebanon; mh354@aub.edu.lb (M.H.); nr50@aub.edu.lb (N.R.); gb02@aub.edu.lb (G.B.)

**Keywords:** tomosynthesis, biopsy, mammography, breast lesion, troubleshooting

## Abstract

Since its introduction, digital breast tomosynthesis (DBT) has been widely incorporated in screening for breast cancer due to its lesser recall and higher cancer detection rates. Some screen-detected lesions may be visible only by DBT, requiring biopsy using DBT guidance. This review article dissects the different steps of tomosynthesis-guided biopsy and discusses the different obstacles that might be encountered during each step while providing the appropriate solutions, hence allowing physicians to perform a successful biopsy with the least patient discomfort.

## 1. Introduction

Digital breast tomosynthesis (DBT) was introduced to clinical practice after FDA approval in 2011. It was widely incorporated in the screening setting, where it is performed either in combination with full-field digital mammography (FFDM) or alone with the reconstruction of synthetic mammograms [1]. DBT is superior to FFDM in detecting breast cancer while reducing recall rates [2,3]. When lesions are identified on screening DBT, the patients are recalled, the additional workup of spot compression views and targeted ultrasound is performed, and the appropriate BI-RADS category is assigned. Although the absence of an ultrasound correlate frequently indicates a non-malignant etiology [1,4], the positive predictive value of biopsies performed for DBT-detected lesions occult on both 2D mammography and ultrasound reaches up to 26% [5]. These BI-RADS 4 or 5 DBT-detected lesions can only be sampled with DBT guidance. As such, mastering DBT-guided biopsy has become very important to breast radiologists.

The procedure shares numerous common steps with conventional stereotactic guided biopsy; the details of the procedure itself and the consecutive steps to follow have been previously extensively described [6,7]. However, DBT-guided biopsy poses some unique challenges related to the tomosynthesis technique and the confirmation of adequate sampling of non-calcified lesions. This article intends to discuss the consecutive technical challenges that radiologists might encounter while performing this procedure (Table 1).

## 2. The Procedure

### 2.1. When Should I Recommend a DBT-Guided Biopsy?

If a lesion identified by DBT shows a clear correlate on ultrasound, then an ultrasound-guided biopsy would be the preferred next step, as it usually is the quickest, safest, most comfortable, and least expensive breast biopsy modality [8]. Ultrasound-guided biopsy allows free needle control and multidirectional access sites with no exposure to ionizing radiation [8].

If a lesion shows no sonographic correlate and can be identified by 2D mammography, a stereotactic guided biopsy can be performed. However, compared to the traditional stereotactic guided biopsy, DBT-guided biopsy offers the additional advantages of reduced radiation exposure [9,10], reduced procedure time, a higher success rate, and improved lesion visibility [9,11]. As such, DBT-guided vacuum-assisted biopsy can be used to sample exclusively DBT-identifiable lesions and all ultrasound-occult lesions visible on 2D mammography [9,12]. Vacuum-assisted biopsies are associated with an extremely low risk of complications [13].

An MRI-guided biopsy provides the main advantage of adequate sampling of lesions that are very subtle or not identifiable on conventional imaging. It is, however, a more time-consuming procedure that is less well tolerated by the patients, as it requires the patient to hold still in a prone position with the breast compressed for about 40–60 min and is usually the most expensive procedure. Moreover, the limited accessibility inherent to this procedure may prevent adequate sampling of lesions in extreme locations [8].

### 2.2. Patient Preparation

It is crucial to explain to the patient in detail what she will experience during the biopsy. The patient should also be informed of the position she will be in during the biopsy (supine versus sitting) and asked to maintain steadiness for the entire duration of the biopsy in order not to displace the target. Possible procedural complications should also be discussed.

At the beginning of the procedure, the patient should be seated comfortably and well supported to minimize motion. The breast is compressed according to the planning performed ahead of time. The compression need not be painful but should be strong enough to stabilize the breast and avoid motion. Subsequently, a tomosynthesis image is obtained, and the area of concern is identified. The target is placed at the tomosynthesis slice showing the lesion most clearly. The needle to be used is specified on the software, and the accessibility of the lesion is confirmed. An anesthetic medication is then injected, usually lidocaine with or without adrenaline.

### 2.3. How Should I Access the Lesion?

Prone biopsy tables allow one approach in which the biopsy needle is perpendicular to the breast platform. Upright tables offer two approaches, parallel and perpendicular, that can be used interchangeably depending on lesion location, accessibility, and radiologist preference. Table 2 summarizes the advantages and inconveniences of each.

#### 2.3.1. Needle Perpendicular to the Breast Platform

**Figure 1:**
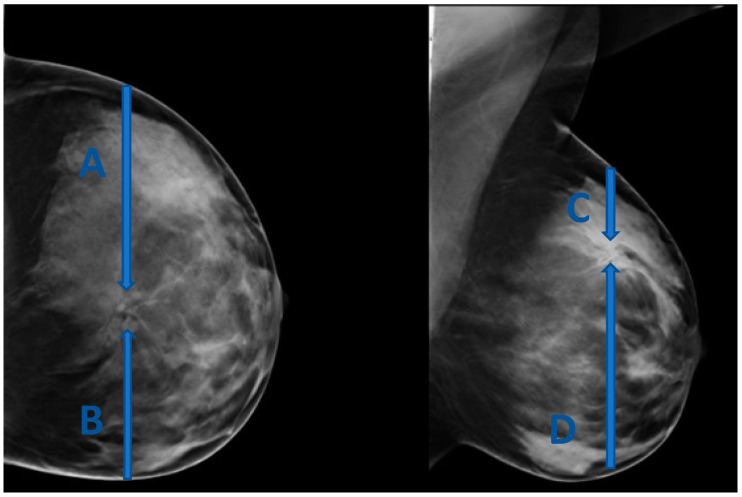
(**A**–**D**) represent four possible approaches for biopsy of a lesion located in the upper inner aspect of the breast. (**C**) is the shortest skin-to-lesion route, so a craniocaudal approach should be used.

The site of entry in this approach should be determined based on the location of the lesion dictating the shortest skin-to-lesion route. For example, when the lesion is closest to the superior aspect of the breast, a cranio-caudal (CC) approach is favored (Figure 1).

**Figure 2:**
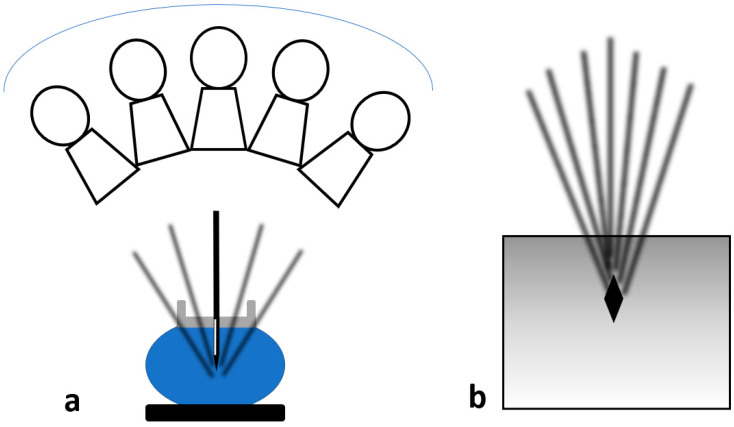
When the needle is in a perpendicular approach, as the tube is rotating along its course between −15 and +15°, each slice obtained will show the different projections of the needle depending on the tube location (**a**); the result will be multiple dense lines converging towards the tip of the needle (black diamond) projecting on the confirmation images obtained after insertion of the needle (**b**).

**Figure 3:**
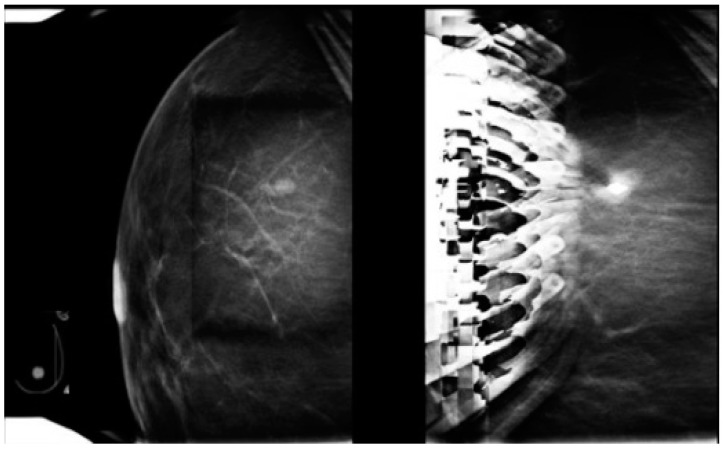
Artifact resulting from the orientation of the biopsy needle perpendicular to the breast platform. The lesion is large enough to remain visible, unaltered by the artifact on the post-fire tomosynthesis image.

**Figure 4:**
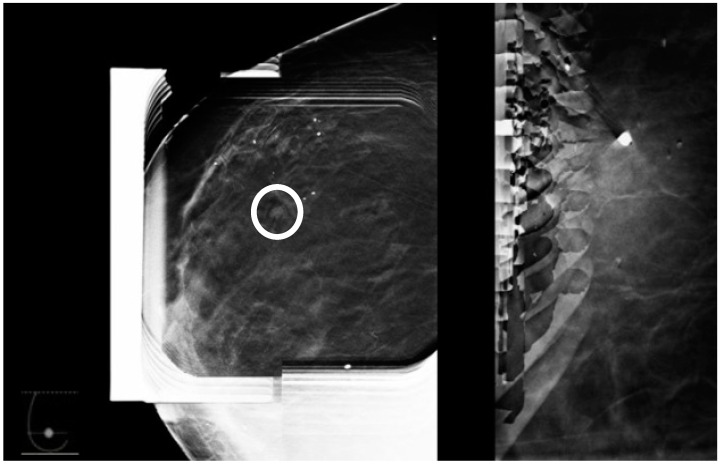
The lesion detected on the planning tomosynthesis image (circle) is masked by the artifact on the post-fire image.

This is the most frequently used approach and allows easy access to the skin entry site through the fenestrated compression paddle. This needle orientation will however cause an artifact on the tomosynthesis images (Figure 2) that may potentially significantly limit the ability to visualize the target and ensure adequate post-fire needle position (Figure 3 and Figure 4). Obtaining the confirmation images in 2D may help bypass this artifact; however, in case the abnormality is subtle or small, 2D images may be less able to clearly demonstrate the lesion.

**Table 2:** Advantages and difficulties of the perpendicular and parallel approach.

	Advantages	Difficulties
Needle perpendicular to breast platform	Most frequently used Easy access to skin entry site	Needle artifact that may impede the visibility of the target
Needle parallel to breast platform	Eliminates needle artifact Useful in patients with small/thin breasts Can be performed with a non-fenestrated paddle	Difficult access to skin entry site An appropriate compression paddle should be used

#### 2.3.2. Needle Parallel to the Breast Platform

In this approach, the breast is compressed in the CC position, and lesions are accessed horizontally from medial or lateral depending on their location. The breast can also be compressed in the mediolateral (ML) or lateromedial (LM) position, and the lesion can be accessed from the cranial or from the caudal aspect of the breast.

**Figure 5:**
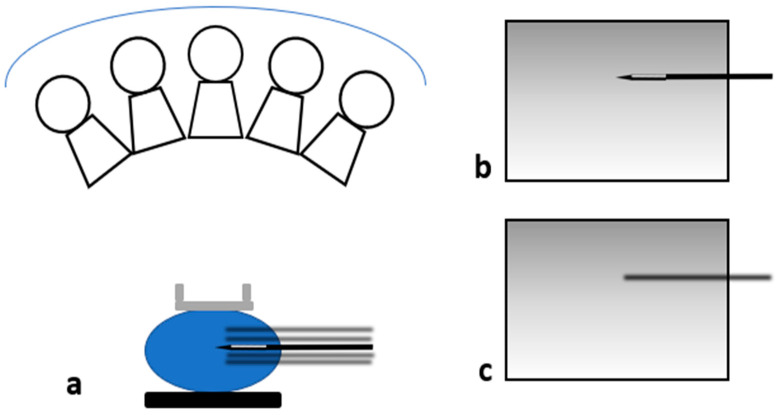
When the needle is in a parallel approach, as the tube is rotating (**a**), the needle will either appear sharp when it is in the plane being imaged (**b**) or blurred when it is not (**c**).

**Figure 6:**
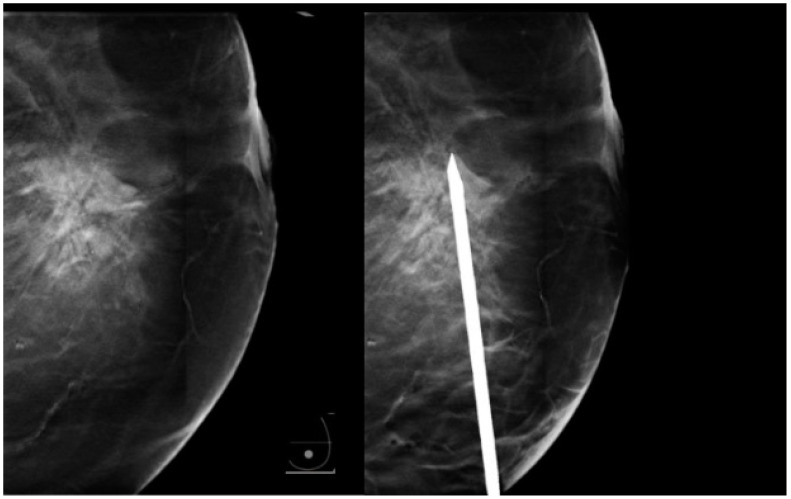
Biopsy needle parallel to the platform, avoiding the previously described artifact.

**Figure 7:**
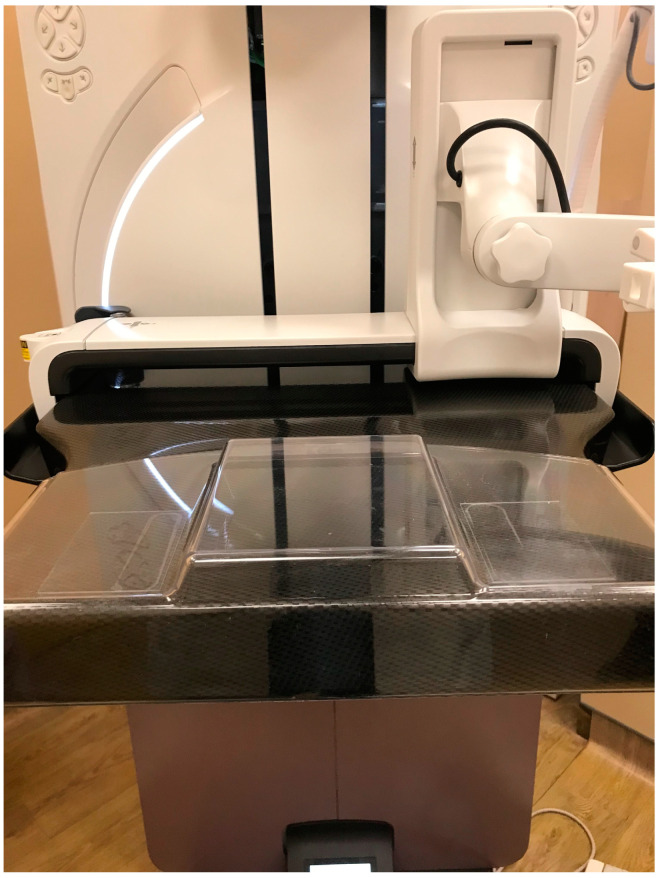
A spacer (arrows) can be positioned on the breast platform to allow elevation of the breast.

In this approach, the needle artifact described earlier is not present, and the visibility of the target is maintained (Figure 5 and Figure 6), but access to the skin entry site on the breast might be more difficult. Depending on the vendor, this approach could be performed with a non-fenestrated paddle, allowing better compression of the breast and improved visibility of subtle lesions. This access can prove especially useful in patients with small breasts where the breast thickness after compression will not allow a safe stroke margin, defined as the space between the needle tip and the breast platform and conventionally set at 3–5 mm. In slender breasts, a biopsy in this approach may still be unsuccessful in case the breast’s thickness is slim and the plastic handle of the needle is colliding against the breast platform. In this case, and depending on the vendors, a spacer can be inserted to elevate the breast from the platform and allow the successful introduction of the needle (Figure 7). Alternatively, gauze or other materials can be used to serve the same purpose.

#### 2.3.3. Compression Paddles and Changing Access

The fenestrated compression paddles are available with either a right-sided or a left-sided arm. If a perpendicular approach is planned, any paddle can be used. However, if a parallel approach will be performed, the appropriate paddle should be chosen according to the site of access planned by the radiologist. For example, if the lesion to be biopsied is located laterally in the left breast, a compression paddle with a right-sided arm should be used.

**Figure 8:**
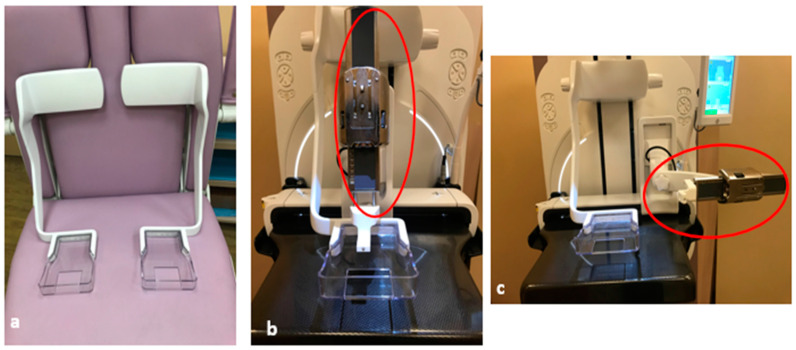
Compression paddles are available with a left-sided or right-sided arm (**a**). The appropriate sidearm should be used in case shifting from a perpendicular approach (**b**) to a parallel approach (**c**) might be needed in order to allow mobilization of the needle holder (red oval) while maintaining the breast in the same compression.

If deemed necessary during the procedure, a perpendicular approach can always be switched to a parallel approach while maintaining breast compression. Care should be taken to prospectively use the appropriate compression paddle depending on the site of access to avoid repeating the planning image. (Figure 8).

### 2.4. Why Can I Not See the Lesion on the Planning Mammogram?

After the approach has been planned and the patient is positioned, planning 3D images are obtained to localize the target. Occasionally, the lesion of concern may not be visible on the planning mammograms [14,15].

This may happen if a significant air gap is present surrounding the breast, which may decrease the quality of the images obtained. It may be helpful in such cases to fill any gap with a malleable putty [7].

In addition, some faint calcifications seen on FFDM may not be identified on the stereo images, as these have less spatial and contrast resolution [14]. Using a low kilovoltage of around 25 Kvp and preventing motion by performing adequate compression may improve visibility [16].

This may also happen if the target is in an extreme location posteriorly, medially, or laterally. The radiologist should then re-evaluate the diagnostic mammograms and have a thorough understanding of the location of the lesion in the breast. If a planned approach does not demonstrate the lesion, the radiologist can decide to shift to another approach depending on the lesion location. The use of upright tables does offer an advantage in such cases as it allows more flexibility in patient positioning and increased ability to reach lesions in challenging locations, using, for example, cleavage and axillary tail views.

If a different approach is attempted and is also unsuccessful, it may be helpful to image the breast using an alphanumeric grid and mark the skin overlying the lesion, then re-attempt the biopsy.

Although DBT-guided biopsy is associated with a higher biopsy success rate than conventional stereotactic-guided biopsy [9], the lesion may rarely remain invisible during the procedure. In this case, the biopsy should be canceled, and these lesions will need to undergo either excisional biopsy, short-term follow-up, or problem-solving MRI depending on the type of the initial lesion (calcifications, mass, architectural distortion, or asymmetry) and the degree of clinical suspicion.

### 2.5. I Can See the Lesion, Why Is My System Not Allowing Me to Proceed?

**Figure 9:**
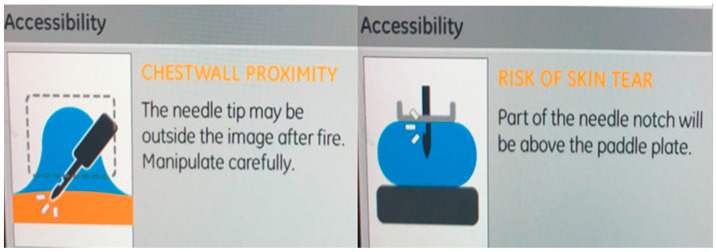
Examples of warning messages in cases of lesions at extreme locations.

**Figure 10:**
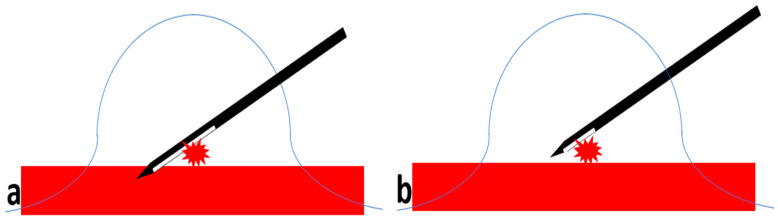
(**a**) In lesions very deeply seated, the use of a needle with a long aperture might carry the risk of chest wall injury (red rectangle). (**b**) A needle with a smaller aperture will allow successful sampling of such lesions.

After the lesion is visualized on the planning images, the target location marked and the needle chosen, the software calculates the *x*, *y*, and *z* coordinates of the lesion. Occasionally, the system may not allow proceeding with the biopsy when any of the coordinates is deemed to pose a risk to the patient. This may happen, for example, in thin breasts not allowing a safe stroke margin, in lesions superficially located where there is a risk of skin injury [17,18] or lesions deeply located carrying a risk of chest wall injury [18] (Figure 9). In these cases, the following might be helpful:

- Create a lidocaine wheel at the skin entry site to displace the lesion from the overlying skin.
- Change the needle used for one with a smaller gauge or shorter aperture to decrease the risk of injury (Figure 10). Additional plastic aperture sleeves may be used to further decrease the needle trough and minimize the risk of injury [7].
- Change the approach, even if this means not using the shortest route.
- Decrease breast compression, and if needed, roll/push the breast tissue in a manner to manually increase the thickness at the area of concern.
- Create a target offset or target a safe, accessible edge of the lesion (for example, the anterior edge in very posteriorly located lesions) or an adjacent landmark and then perform directional sampling from the appropriate clock face.

Despite these maneuvers, some lesions will remain inaccessible [16] by a percutaneous biopsy. These should undergo either excisional biopsy, short-term follow-up, or problem-solving MRI as discussed earlier.

### 2.6. Is My Needle at the Level of the Lesion?

**Figure 11:**
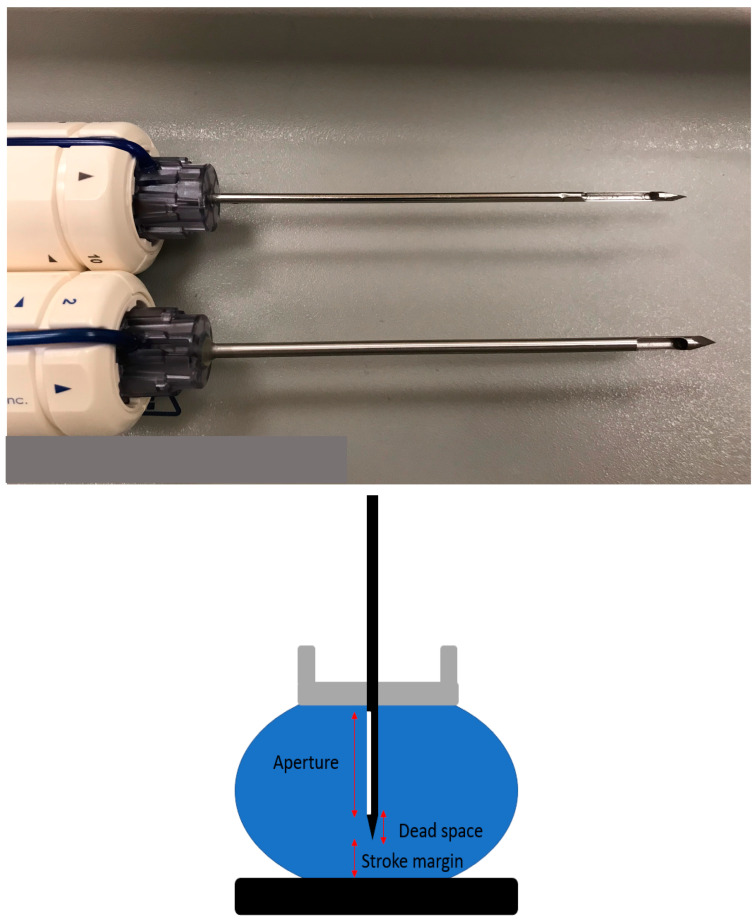
A close-up view showing two different needles, one 12 gauge and 20 mm aperture (**top**) and the second 9 gauge and 12 mm aperture (**bottom**). Note the clock face annotation on the plastic handle; the small arrowhead indicates the 12 o’clock position. The aperture is the area of the needle where sampling takes place. The metallic component distal to the aperture is the dead space. The stroke margin is the distance between the tip of the needle and the edge of the breast.

Figure 11 shows an image of the metallic part of a vacuum biopsy needle. The aperture where sampling takes place is located at the distal aspect and is followed by a short dead space. All needles have the clock face indicated on them, allowing the radiologist to identify the location being sampled. Irrespective of the approach, the needle is always advanced with the 12 o’clock position oriented towards the chest wall.

After the needle has been fired or manually inserted, 2D or 3D images are obtained to confirm adequate position of the needle with its aperture at the level of the target.

Occasionally, and more likely in dense breasts, the needle may push and displace the tissues rather than penetrate through them [19], resulting in an inadequate location of the needle with respect to the lesion. The radiologist should carefully assess the relation of the lesion and the needle to determine whether the lesion is close enough to the needle to be accessible and its exact location with respect to the needle to determine the clock face where directional sampling is needed. If the lesion is close enough, the needle position can be modified manually along any or a combination of the *x*, *y,* or *z* axes to reach the lesion. Otherwise, the needle should be withdrawn, and retargeting performed. A new skin incision is frequently not needed, as the needle tip can be placed at the initial incision and then the needle dialed to the new target.

Assessment of the location of the target with respect to the lesion will differ depending on the approach used.

#### 2.6.1. In the Perpendicular Approach

**Figure 12:**
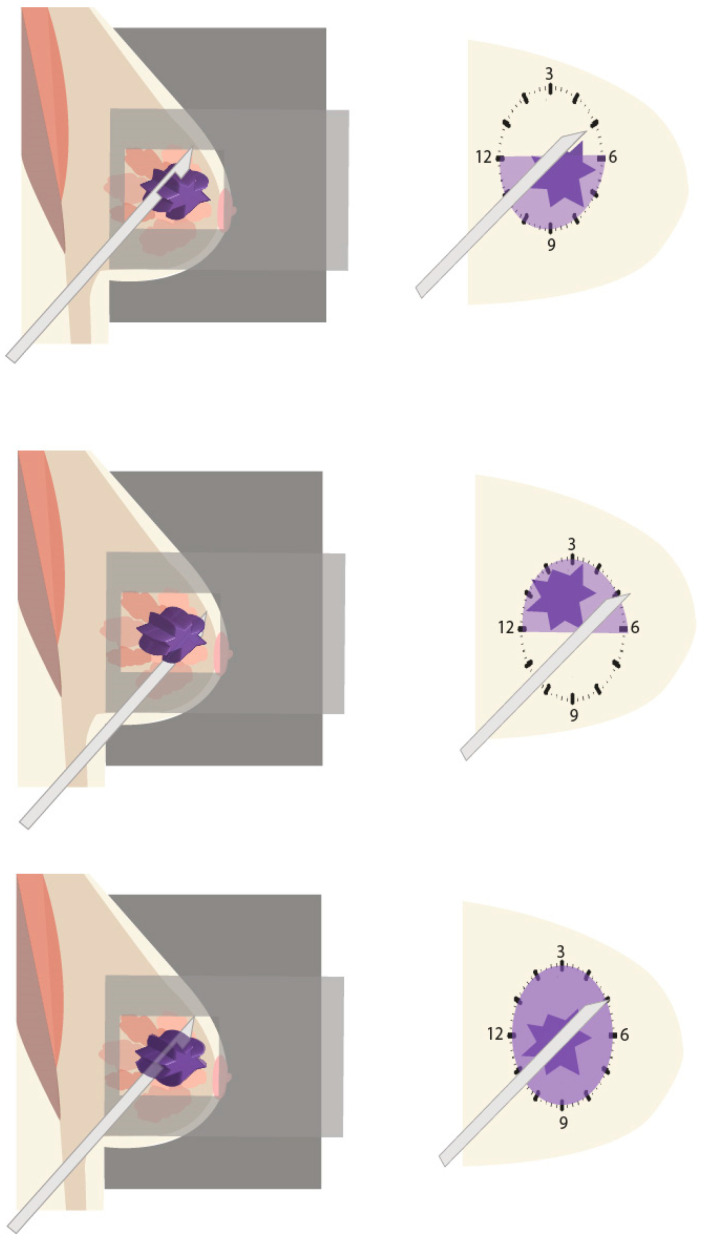
Different scenarios illustrating how to predict in a perpendicular approach the location of the needle with respect to the lesion and the clock face where directional sampling needs to take place. **Upper row**: The lesion is identified inferior to the needle; hence directional sampling needs to be obtained from the shaded quadrants from 6 to 12 o’clock through 9 o’clock. **Middle row**: The lesion is identified superior to the needle; hence directional sampling needs to be obtained from the shaded quadrants from 12 to 6 o’clock through 3 o’clock. **Lower row**: The needle penetrates the lesion; circumferential tissue sampling is feasible.

If DBT guidance is used, the artifact created by the metallic needle may limit the visibility of the lesion. Landmarks will in such cases prove very helpful. It is important to ensure that the tip of the needle is distal to the lesion and to check in which clock face the target is located. This is usually easy to evaluate as the needle and the target are present together in the same image. Figure 12 demonstrates different scenarios illustrating how to assess in a perpendicular approach the location of the needle with respect to the lesion and the clock face where directional sampling needs to take place.

#### 2.6.2. In the Parallel Approach

**Figure 13:**
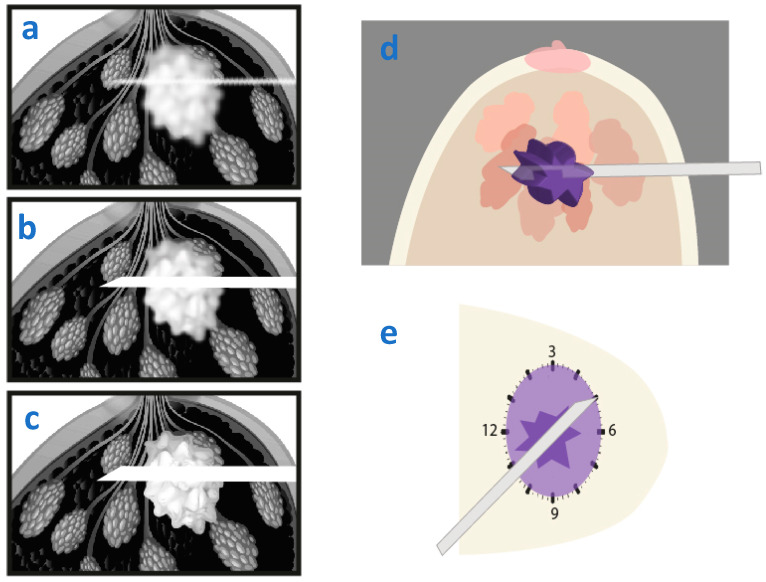
Different scenarios illustrating how to predict the location of the needle with respect to the lesion and the clock face where sampling needs to take place based on the tomosynthesis images. (**a**–**c**) represent post-fire tomosynthesis images from cranial to caudal, (**d**) illustrates the expected location of the lesion with respect to the needle, (**e**) illustrates the lesion and the shaded quadrants where sampling needs to be directed. In this scenario, the needle and lesion both become sharp at the same slice (**c**), indicating they are both at the same level. The needle projects in the center of the lesion. The needle is hence perfectly positioned at the center of the lesion (**e**), and sampling can be directed to all the quadrants on the clock face.

**Figure 14:**
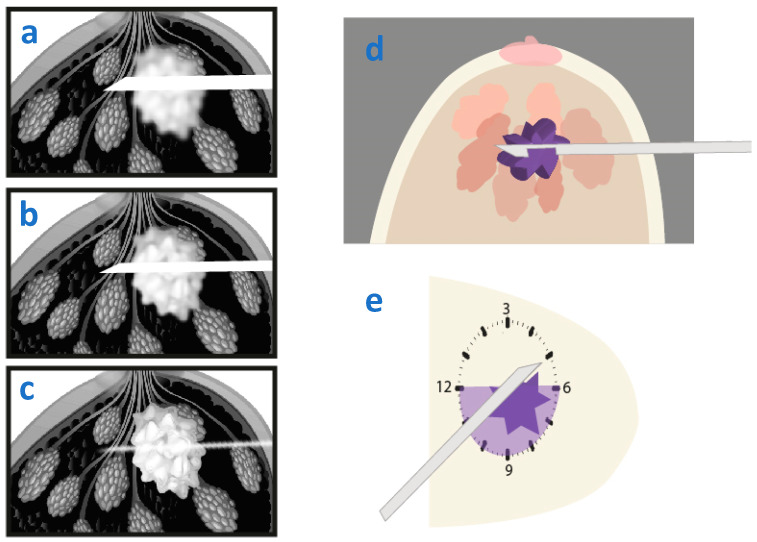
The needle and the lesion are not seen as sharp at the same level. Instead, the needle appears sharper at a more cranial slice level (**a**) than the lesion (**c**), (**b**) being an intermediate slice level between the two showing both the needle and the lesion not sharp. The needle projects over the lesion. Based on this, the expected location of the needle would be superior with respect to the lesion, as depicted in (**d**). Sampling should hence be directed at the shaded quadrants 6 to 12 going through 9 on the clock face (**e**).

The needle artifact is eliminated but evaluating the clock face location of the target might prove more challenging when the needle ends up in a different plane, i.e., more superior or inferior. Determining the location of the needle and target will hence depend not only on the projection of the lesion with respect to the needle, but also on the slice level that shows each the sharpest. Figure 13 and Figure 14 demonstrate two possible different scenarios illustrating how to predict in a parallel approach the location of the needle with respect to the lesion and the clock face where directional sampling needs to take place based on the tomosynthesis images.

In dense breasts where the needle might not penetrate the tissues properly, it might be helpful—if allowed by the size of the breast—to advance the needle an additional centimeter distal to the target location to create a tract, then withdraw back to the final location.

### 2.7. Why Is My Clip Marker Not Deploying?

At the end of the procedure, the clip marker is inserted, the biopsy needle is slightly withdrawn, and radiographs are obtained while the breast is held in compression to ensure the clip has been deployed.

Occasionally, the clip marker can remain stuck to the needle tip, often because of retained tissue fragments or blood clots [20]. This can be prevented by avoiding an unnecessarily large biopsy cavity that carries a higher risk of hematoma formation and by performing a good lavage and aspiration post biopsy to remove blood clots as much as possible.

Should the clip marker get stuck to the needle tip, the radiologist can attempt push injecting 1–2 cc of saline to free the clip; this does, however, carry the risk of clip displacement. Alternatively, the needle may be removed while taking care not to lose the clip at its tip; the tip can be cleaned of any adherent blood clots/tissue fragments, and then the needle reinserted and clip marker deployed.

### 2.8. My Clip Marker Is Not Where It Should Be!

**Figure 15:**
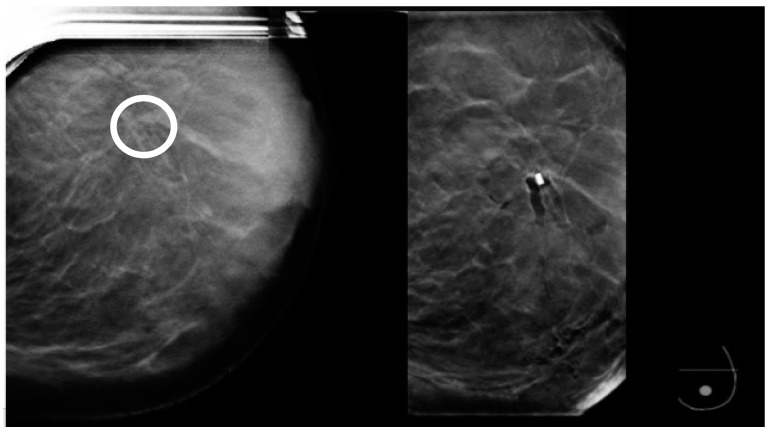
**Left**: prebiopsy image showing a suspicious distortion (circle). **Right**: The biopsy cavity and clip marker are both centered at the lesion.

**Figure 16:**
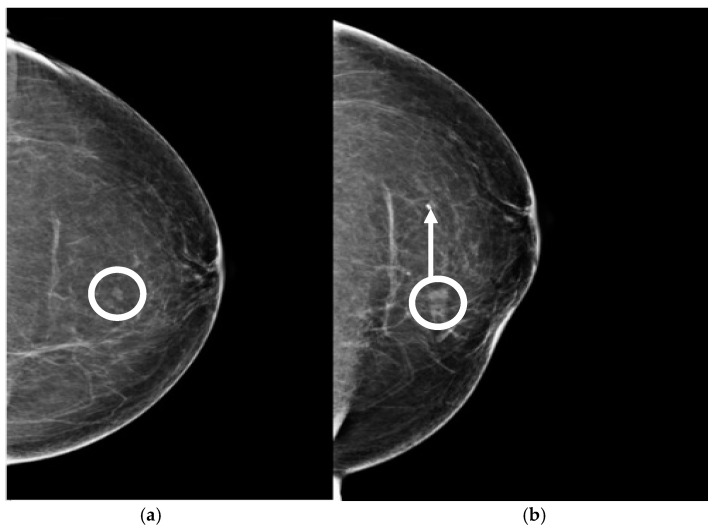
(**a**) Pre-biopsy mammogram revealing a mass in the inner quadrant of the left breast. (**b**) Post-biopsy mammograms revealing a cavity centered at the lesion (circle) but lateral displacement of the clip marker (arrow).

After insertion of the clip marker, the biopsy needle is slightly withdrawn, and documentation images are taken while the patient’s breast is compressed to ensure adequate clip deployment, and then post-procedure mammograms are obtained to ensure proper placement of the clip (Figure 15). Occasionally, the clip marker gets displaced from the target site (Figure 16). The rate of clip migration more than 1 cm varies between 28% [21] and 13.1% [22]. Displacement happens more frequently in medially located lesions [22], predominantly fatty breast [22,23], and patients who develop hematomas [24]. As such, clip displacement can be prevented by minimizing hematoma formation and slowly decompressing the breast to avoid the accordion effect that will cause displacement at the time of releasing the patient’s breast [24].

**Figure 17:**
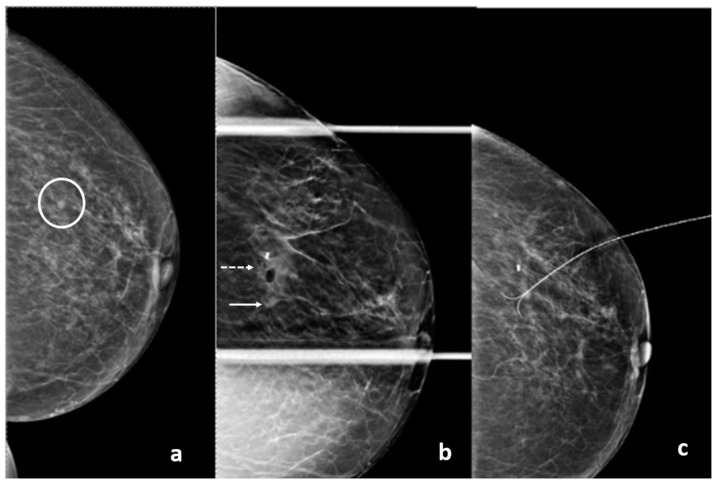
(**a**) A 65-year-old patient with a screen-detected left breast mass (circle). (**b**) Post-procedure mammograms after DBT-guided biopsy show the biopsy cavity and clip marker (dashed arrow) to be laterally located with respect to the mass (arrow). Pathology shows atypical ductal hyperplasia. Because of concern about proper sampling and radiological-pathological discordance, a surgical excision after wire localization was performed (**c**). Final pathology reveals a grade 1 invasive ductal carcinoma.

Should the clip marker get displaced, the radiologist should carefully assess the location of the clip, biopsy cavity, and target lesion with respect to each other to differentiate clip migration from inadequate targeting. Adequate sampling is ensured by documenting the presence of calcifications within the cores in case of lesions with calcifications or demonstrating the superposition of the lesion and biopsy cavity on the post-procedure mammograms in case of lesions with no calcifications. If the radiologist is satisfied with the sampling, the clip displacement must be mentioned in the report, and the location of the clip marker with respect to the target site is clearly stated [22,24]. At our practice, if the radiologist is unsure about the appropriateness of the sampling, we wait for the pathology results and carefully assess radiological-pathological concordance to determine whether targeting was adequate and accordingly decide whether any further action, such as repeat biopsy or surgical excision, needs to be considered (Figure 17).

### 2.9. Post-Procedure Care

Following deployment of the clip marker, the breast is slowly decompressed, and compression is applied for 5–10 min to ensure hemostasis. Ice packs may be applied as well. The incision is usually small enough not to warrant any sutures, and post-procedure antibiotics are not indicated. Post-procedure pain is managed with analgesics; the patient is instructed to avoid NSAIDs due to their anti-coagulant properties. The patient is also informed to avoid heavy weightlifting and coming close to heat sources to minimize the risk of bleeding and to avoid exposing the wound to water to avoid infection. The patient is also instructed on how to follow up with her results. At our facility, the referring physician provides the patient with the pathology results in 4–5 working days.

## 3. Conclusions

With the increasing use of DBT-guided breast biopsies, radiologists ought to become familiar with the technique used and the challenges that might be encountered during the procedure. This troubleshooting guide is intended to become a tool to enhance radiologists’ preparedness and confidence, resulting in successful biopsies and greater patient comfort.

## Figures and Tables

**Table 1:** Summary of the different technical challenges that radiologists may encounter while performing a DBT-guided biopsy, the possible underlying causes, and steps that can be taken to overcome them.

Problem	Possible Causes	What to Do
Lesion not seen on the planning images	Air gap surrounding the breast Lesion subtle and low contrast Lesion in extreme location in the breast	Fill gap with malleable putty Change compression from CC to lateral and vice versa Use low Kv technique Keep breast well compressed to prevent motion Perform dedicated positioning (cleavage, axilla, etc.) Re-image with an alphanumeric grid to mark the lesion
Lesion seen but not accessible	Thin breast Lesion too superficial Lesion too deep	Create a lidocaine/saline wheel Use a smaller gauge/smaller aperture needle Use a plastic aperture sleeve Decrease breast compression Roll/push breast tissue to increase thickness Change compression from CC to lateral or vice versa Change approach from perpendicular to parallel or vice versa Use a spacer to elevate the breast Target a safe, accessible edge of the lesion and perform a directional sampling
Clip marker not deploying	Clip adherent to residual tissue fragments/blood clot	Perform adequate lavage with saline Perform adequate aspiration to empty biopsy cavity Push inject 1–2 cc of saline to free clip marker Withdraw the needle, clean its tip, and re-attempt deployment
Clip marker displaced	Fatty breast Large biopsy cavity/hematoma formation Accordion effect Medially located lesions	Slowly decompress the breast Ensure adequate sampling was performed Document location of clip with respect to biopsy cavity
